# Pharmacologic Blockade of 15-PGDH Protects Against Acute Renal Injury Induced by LPS in Mice

**DOI:** 10.3389/fphys.2020.00138

**Published:** 2020-03-13

**Authors:** Shuying Miao, Caihong Lv, Ying Liu, Jie Zhao, Ting Li, Chunjiang Wang, Yunfei Xu, Xiaoli Wang, Xianzhong Xiao, Huali Zhang

**Affiliations:** ^1^Department of Pathophysiology, Xiangya School of Medicine, Central South University, Changsha, China; ^2^Department of Pathology, Nanjing Drum Tower Hospital, Nanjing University Medical School, Nanjing, China; ^3^Sepsis Translational Medicine Key Lab of Hunan Province, Changsha, China; ^4^Department of Neurosurgery, Xiangya Hospital, Central South University, Changsha, China; ^5^Department of Physiology, Changzhi Medical College, Changzhi, China; ^6^Department of Pharmacy, The Third Xiangya Hospital, Central South University, Changsha, China; ^7^Department of Pathology and Pathophysiology, Jishou University, Jishou, China

**Keywords:** AKI, 15-PGDH, LPS, autophagy, apoptosis, oxidative stress

## Abstract

Prostaglandin pathway plays multiple roles in various physiological and pathological conditions. The present study aimed to investigate the effect of 15-hydroxyprostaglandin dehydrogenase (15-PGDH), a key enzyme in the degradation of prostaglandins, on lipopolysaccharide (LPS)-induced acute kidney injury (AKI) in mice. In this study, male C57BL/6J mice were injected intraperitoneally with LPS (10 mg/kg). SW033291, a potent small-molecule inhibitor of 15-PGDH, was used to investigate the therapeutic potential of 15-PGDH inhibition on LPS-induced AKI. We discovered that the expression of 15-PGDH protein was upregulated in kidneys of LPS-stimulated mice, and it was mainly localized in the cytoplasm of renal tubular epithelial cells in renal cortex and outer medulla. SW033291 administration improved the survival rates of mice and attenuated renal injury of mice that were challenged by LPS. Additionally, inhibition of 15-PGDH also reversed LPS-induced apoptosis of renal cells, increased expression of anti-apoptotic protein Bcl-2, and downregulated expression of Fas, caspase-3, and caspase-8. Pretreatment of SW033291 enhanced autophagy in kidney cells after LPS stimulation. Our data also showed that inhibition of 15-PGDH relieved the level of lipid peroxidation and downregulated NADPH oxidase subunits induced by LPS in mice kidneys but had no significant effect on the release of inflammatory factors, such as IL-6, IL-1β, TNF-α, and MCP-1. Our study demonstrated that inhibition of 15-PGDH could alleviate LPS-induced AKI by regulating the apoptosis, autophagy, and oxidative stress rather than inflammation in mice.

## Introduction

Acute kidney injury (AKI) is one of the most common and serious complications of sepsis with a high incidence and mortality rate. Sepsis accounts for up to 50% of cases of AKI in intensive care unit patients (Alobaidi et al., 2015). Mortality in patients with septic AKI is approximately doubled when compared with patients with sepsis alone (Jaworska et al., 2015; Thomas et al., 2015). However, little progress has been made toward the treatment of septic AKI in recent decades (Prowle, 2018). This may be related to the complex pathophysiological mechanism of septic AKI. Therefore, it is critical to further elucidate the mechanism of septic AKI.

It has been suggested that apoptosis, autophagy, oxidative damage, and inflammatory response are all involved in the development of AKI. Apoptosis and autophagy are common processes that maintain cellular metabolism and tissue homeostasis. A large number of studies show that renal tubular epithelial cells undergoing apoptosis can promote the development of renal dysfunction in ischemia-reperfusion (I/R) and septic AKI model (Lerolle et al., 2010). Autophagy is activated to prevent renal dysfunction from injury (Song et al., 2018). Studies have shown that renal tubular epithelium is the main site of oxidative stress, and reactive oxygen species (ROS) and reactive nitrogen (RNS) play important roles in acute renal tubular injury during sepsis (Pathak et al., 2012). In addition, systemic inflammatory response is closely related to septic AKI (Frei et al., 2010). When LPS enters the bloodstream, it forms a LPS/LBP/CD14 complex with lipopolysaccharide binding protein (LBP) and CD14, which in turn binds to toll-like receptor 4 (TLR-4) and activates the LPS signaling pathway, promoting the release of inflammatory factors, chemokines, and immune cell infiltration (Wu H. et al., 2007; Areschoug and Gordon, 2018).

15-hydroxyprostaglandin dehydrogenase (15-PGDH), a key enzyme in the degradation of prostaglandins, including prostaglandin E_2_ (PGE_2_), can catalyze the conversion of 15-hydroxy group of PGE_2_ into a 15-keto group to produce a biologically inactive PG and antagonize the function of prostaglandin synthase COX-2 (Tai, 2011). Most previous studies have focused on the roles of COX-2 and its downstream prostaglandins, but there have been few studies on 15-PGDH. Recent studies have shown that 15-PGDH plays an important role in the development of inflammation-related diseases. In rheumatoid arthritis (RA), hydroxychloroquine can induce 15-PGDH expression through the MAP kinase pathway, while the exact role of PGDH as a potential target for RA treatment remains to be elucidated (Kim et al., 2015). Zhang et al. (2015) found that inhibition of 15-PGDH by selective inhibitor SW033291 or knockdown of 15-PGDH can promote the recovery of hematopoietic function. However, the biological function of 15-PGDH inhibition in AKI has not yet been reported. Therefore, we investigated the effect of 15-PGDH on AKI induced by lipopolysaccharide (LPS) in the hopes of determining whether 15-PGDH exerts its role by regulating information, autophagy, apoptosis, and oxidative stress. Our study provides new insights into the understanding on the pathogenesis and therapy of AKI caused by sepsis.

## Materials and Methods

### Animals

C57BL/6J male mice aged 8–10 weeks (20–25 g) were purchased from Hunan Sleek Jingda Experimental Animal Co., Ltd. Mice were housed in a temperature-controlled room (25 ± 2°C) with relative humidity of 40∼60% on a 12 h light/dark cycle during the study. All animal experiments were approved by the Experimental Animal Ethics Committee of Central South University. The experimental mice were acclimatized for 7 days before administration of a 10 mg/kg dose of LPS or control saline.

For analysis of 15-PGDH expression in LPS-induced AKI, 12 mice were divided into four groups with three mice in each group (*n* = 3), and each mouse was administered intraperitoneally with 10 mg/kg body weight LPS (from *Escherichia coli* 0111:B4, Sigma-Aldrich, St. Louis, MO, United States) for 0, 6, 12, and 24 h, respectively. Under anesthesia, their eyeballs were removed for collection of blood, and tissues were collected for Western blot and immunohistochemistry.

For effect of SW033291 on inhibiting 15-PGDH, 30 mice were divided into five groups with six mice in each group (*n* = 6), and each mouse was administered intraperitoneally with 10 mg/kg body weight SW033291. Animals were sacrificed at 0, 3, 6, 12, and 24 h, followed by collection of kidneys for PGE_2_ ELISA experiment. SW033291 was diluted according to Zhang et al. (2015).

For survival experiments, 100 mice were divided into four groups (*n* = 20 in control and SW033291 groups; *n* = 43 in LPS group; *n* = 37 in LPS + SW033291 group). Following LPS (10 mg/kg body weight), stimulated mice were treated with 10 mg/kg SW033291 or vehicle control twice daily for five doses. Then, mice survival was monitored and recorded daily. The other 70 mice were used to explore the role and mechanism of 15-PGDH on LPS-induced AKI, as described in the section below.

### Serum and Tissue Samples

The mice were anesthetized, and the blood was collected for the assay of renal function. The kidneys were used for analysis of real-time PCR, Western blot, ELISA, HE staining, and immunohistochemistry.

### Renal Function Test

Scr assay kit (C011-2-1) and BUN assay kit (C013-2-1) were acquired from Nanjing Jiancheng Bioengineering Institute. After the mice were anesthetized, blood was collected from the eyeballs of the mice for serum creatinine (Cr) and urea nitrogen (BUN) assay, according to the manufacturer’s instructions. After sacrificed, the kidneys of each mouse were quickly removed and stored at −80°C.

### Histological Data

After being fixed in 4% paraformaldehyde solution, the kidney tissue was dehydrated, transparent, paraffin embedded, and sliced. Then, HE staining was performed and observed under a microscope. Double-blind method was used to assess the renal tubular injury by the magnitude of tubular epithelial swelling, loss of brush border, interstitial cell infiltration, tubular cell necrosis, vacuolation, and desquamation on the basis of the following scale: grade 0, no morphological deformities; grade 1, 1–25%; grade 2, 26–50%; grade 3, 51–75%; grade 4, 76–100% (Kojima et al., 2007; Havakhah et al., 2014).

### Real-Time PCR

The total RNA from kidney tissue was isolated by using TRIzol. The isolated mRNA was used as a template to synthesize cDNA by using PrimeScriptTM RT Master Mix (Takara, Japan). Real-time PCR was performed by using the One Step SYBR^®^ PrimeScript TM RT-PCR Kit in a Biosystems 7500 instrument. The amplification conditions were 95°C for 30 s, 95°C for 5 s, and 60°C for 34 s for 40 cycles. The primer sequences used were presented as follows (see Table 1).

**TABLE 1:** The sequences of primers.

**Primers**	**Species**	**Forward (5′–3′)**	**Reverse (5′–3′)**
Caspase-3	Mouse	CTGACTGGAAAGCCGAAAC	GGACTGGATGAACCACGAC
Caspase-8	Mouse	GTTCAAAGTGCCCTTCCCTG	GTTCAAAGTGCCCTTCCCTG
iNOS	Mouse	CCACCAGGAGATGTTGAACTATGT	CTGTGGCTCTGACCCGTGAA
p47phox	Mouse	GATGGGAAATAGCCGGTGATA	CTATCTGGGCAAGGCTACGG
p40phox	Mouse	GGAGGAGGCTCTGCTGACTG	GTCCTCGTCCTCGGGAAAGT
gp91phox	Mouse	GTTCAAAGTGCCCTTCCCTG	TTGGCTGGGATCACAGGAAT
Catalase	Mouse	ATGGTAACTGGGATCTTGTGG	TTCAGGTGAGTCTGTGGGTT
SOD1	Mouse	CTGTACCAGTGCAGGACCTCA	CACCTTTGCCCAAGTCATCT
Fas	Mouse	GATCTGGGCTGTCCTGCCTCT	TTCACGAACCCGCCTCCTC
bcl2	Mouse	TTGTAATTCATCTGCCGCCG	AATGAATCGGGAGTTGGGGT
β-actin	Mouse	CATTGCTGACAGGATGCAGAAGG	TGCTGGAAGGTGGACAGTGAGG
15-PGDH	Mouse	CCAAGGTAG-CATTGGTGGAT	CCACATCACACTGGACGAAC

### Immunohistochemistry

The expressions of 15-PGDH in the kidneys were detected by immunohistochemistry. The antibody against 15-PGDH antibody (Cayman chemical, Item No.160615) was diluted at 1:100, and the experiment was manipulated according to the instructions of the EnVisionTM FLEX Mini Kit (Dako, K8024). Immunohistochemistry stained tissue sections were scored by two pathologists blinded to clinical and pathological data. According to the dyeing intensity: 0, no staining; 1+, weak; 2+, medium intensity; 3+, strong. Then, H-score was calculated according to the following formula: [1 × (% cells 1 +) + 2 × (% cells 2 +) + 3 × (% cells 3 +)] (Kim et al., 2019).

### Western Blot

Proteins from the kidney samples of mice were extracted by sonicating the tissues in 1% PMSF-containing RIPA buffer and were centrifuged at 4°C for 10 min at 12,000 rpm. The protein concentration was determined by BCA method. Based on the protein concentration, 80–100 μg proteins were mixed with one quarter volume of 5× SDS loading buffer, boiled at 95°C for 10 min to denature the proteins. The proteins were subjected to SDS-PAGE and transferred to membrane. The membranes were blocked at room temperature for 1 h, washed with TBS-T (contains 0.1% Tween-20), and incubated with primary antibodies against 15-PGDH (1:1000 dilution, Cayman chemical, Item No.160615), caspase-3 (1:1000 dilution, proteintech, Item No.66470-2-Ig), microtubule-associated protein light chain 3 (LC-3B) (1:1000 dilution, CST, Item No. 3868), p62 (1:1000 dilution, CST, Item No. 5114), and β-actin (1:2000 dilution, Sigma-Aldrich, Item No. A1978), respectively, for overnight at 4°C. The membranes were then washed with TBST and incubated with secondary antibody for 1 h at RT, and signal was developed by adding the ECL luminescence substrate. The signal intensity of each protein was scanned by intensity-scanning software, and relative expression level of protein was calculated.

### MDA Content, and SOD and CAT Activity

The kidney homogenates were prepared. The MDA content and the activities of SOD and CAT were determined according to the manufacturer’s instructions (Nanjing Jiancheng Bioengineering Institute). The absorbance value (OD value) was measured with a microplate reader, and the MDA content and SOD and CAT activities were calculated according to the formula in the instructions.

### Immunofluorescence

Paraffin sections were deparaffinized and rehydrated, further performed for antigen retrieval and blocked with goat serum for 30 min. The section samples were incubated with LC-3B primary antibody (1:100, CST) overnight at 4°C and washed three times with PBS (pH 7.4) at 5 min for each wash. Sections were then incubated with FITC-labeled anti-rabbit secondary antibody (1:400 dilution, MultiSciences) in the dark for 1 h at room temperature and washed three times with PBS (pH 7.4). The samples were counterstained with DAPI, mounted, and imaged under a fluorescence microscope. The nucleus stained with DAPI was blue under UV excitation, and the positive expression was shown as fluorescein-labeled red light.

### TUNEL Assay

The apoptotic levels in the kidneys were measured according to the instruction of the TUNEL apoptosis detection kit (Roche). The percentage of apoptotic cells was calculated according to the number of apoptotic cells/total number of cells in randomly selected 5–10 fields.

### Transmission Electron Microscope

Freshly isolated kidneys were quickly put into fixing buffer and fixed at 4°C for 2–4 h. After this, samples were transferred to Wuhan Goodbio Technology Co., Ltd., prepared as specimens, examined, and photographed under a transmission electron microscope.

### Determination of PGE_2_ Content

Pre-cooled 1 × PBS was added at a ratio of 1:10 to prepare tissue homogenates. The PGE_2_ concentration of the renal tissue homogenate was determined according to the instruction of PGE_2_ ELISA kit (Huamei Biology Company, Wuhan, China).

### Statistical Analysis

GraphPad Prism version 5.02 (GraphPad Prism Software Inc, San Diego, CA, United States) was used for data analysis. All the experiments were repeated at least three times. Data were presented as mean ± SD. Statistical analysis was performed by ANOVA followed by Tukey’s test. For the purpose of survival analysis, Kaplan-Meier method followed by log-rank test was applied to compare the survival rate between groups. A value of *P* < 0.05 was considered to be statistically significant.

## Results

### Regulation of 15-PGDH in the Kidney of LPS-Induced AKI Mice

**FIGURE 1:**
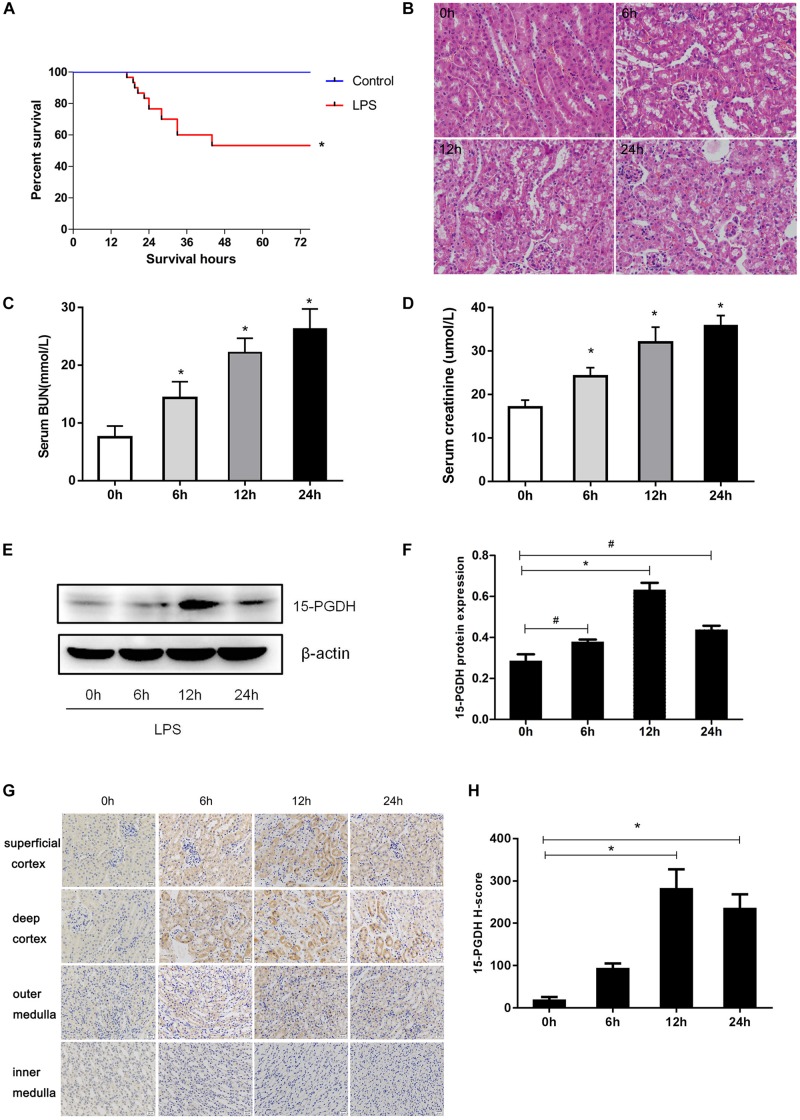
Expression of 15-PGDH in the kidneys of LPS-induced AKI mice model. **(A)** Effect of LPS on the survival of mice (*n* = 30). **(B)** Effect of LPS on renal morphologic changes of mice (200×). **(C)** The levels of serum urea nitrogen (BUN) were measured at different time points (*n* = 6–10). **(D)** The levels of serum creatinine (Cr) were measured at different time points (*n* = 6–10). **(E)** The expression of 15-PGDH in renal tissues at different time points (0, 6, 12, and 24 h) after LPS (10 mg/kg) stimulation (*n* = 3). **(F)** The signal intensity ratio of 15-PGDH to β-actin in renal tissue (*n* = 3). **(G)** Effect of LPS (10 mg/kg) on the expression of 15-PGDH in mouse kidney tissue (200×). **(H)** Comparison of 15-PGDH IHC H-score after LPS (10 mg/kg) stimulation (*n* = 3). Scale = 20 μm. *P < 0.01 (compared with 0 h), ^#^*P* < 0.05 (compared with 0 h). Data are mean ± SD.

At first, we established an AKI mouse model using LPS. As shown in Figure 1A, the survival rate of mice in LPS group significantly decreased to 53% compared to the control group. Through HE staining, we observed marked edema, vacuolar degeneration, inflammatory cell infiltration, and the narrowing of tubular lumen at 12 and 24 h after LPS treatment, contrasting the normal renal morphology in vehicle-treated mice (Figure 1B). Accordingly, the serum levels of creatinine and urea nitrogen were significantly increased after LPS challenge compared to the control mice (Figures 1C,D). We determined the expression of 15-PGDH in kidney in response to LPS treatment. As shown in Figures 1E,F, LPS treatment significantly enhanced protein levels of 15-PGDH in kidneys; the highest level of 15-PGDH occurred at 12 h and lasted for 24 h. By immunohistochemistry, we found that 15-PGDH protein was mainly localized in the cytoplasm of renal tubular epithelial cells in renal cortex and outer medulla and that it had low expression levels in glomeruli and the inner medulla (Figures 1G,H).

### Effect of SW033291 on Inhibiting 15-PGDH in Mice

**FIGURE 2:**
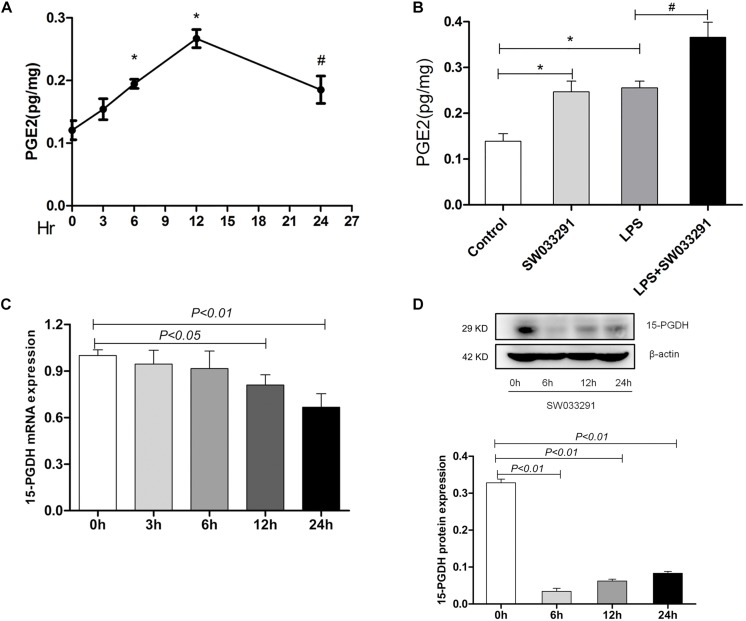
Effect of SW033291 treatment on the blockade of 15-PGDH in the kidneys of mice. **(A)** Effect of SW033291 (10 mg/kg injected twice daily IP for five doses) treatment on renal PGE_2_ content at 0, 3, 6, 12, and 24 h (*n* = 6). *P < 0.01 vs. 0 h, ^#^*P* < 0.05 vs. 0 h. **(B)** PGE_2_ content in kidney tissue of mice induced by LPS (10 mg/kg) at 12 h (*n* ≥ 6).*P < 0.01 vs. Control, ^#^*P* < 0.05 vs. LPS. **(C)** Expression of 15-PGDH mRNA after SW033291 (10 mg/kg) treatment at different time points (*n* = 3). **(D)** Expression of 15-PGDH protein after SW033291 (10 mg/kg) treatment at different time points (*n* = 3). Data are mean ± SD.

As shown in Figure 2A, the PGE_2_ content in kidney time-dependently increased with SW033291 treatment and peaked at 12 h. Strikingly, although the renal PGE_2_ content in both control group and SW033291 group increased after 12 h treatment with LPS, the increase of PGE_2_ content was higher in SW033291 group mice than that in control mice (Figure 2B). Furthermore, the mRNA and protein levels of 15-PGDH were significantly downregulated in kidney after SW033291 treatment (Figures 2C,D).

### SW033291 Treatment Attenuated LPS-Induced AKI in Mice

**FIGURE 3:**
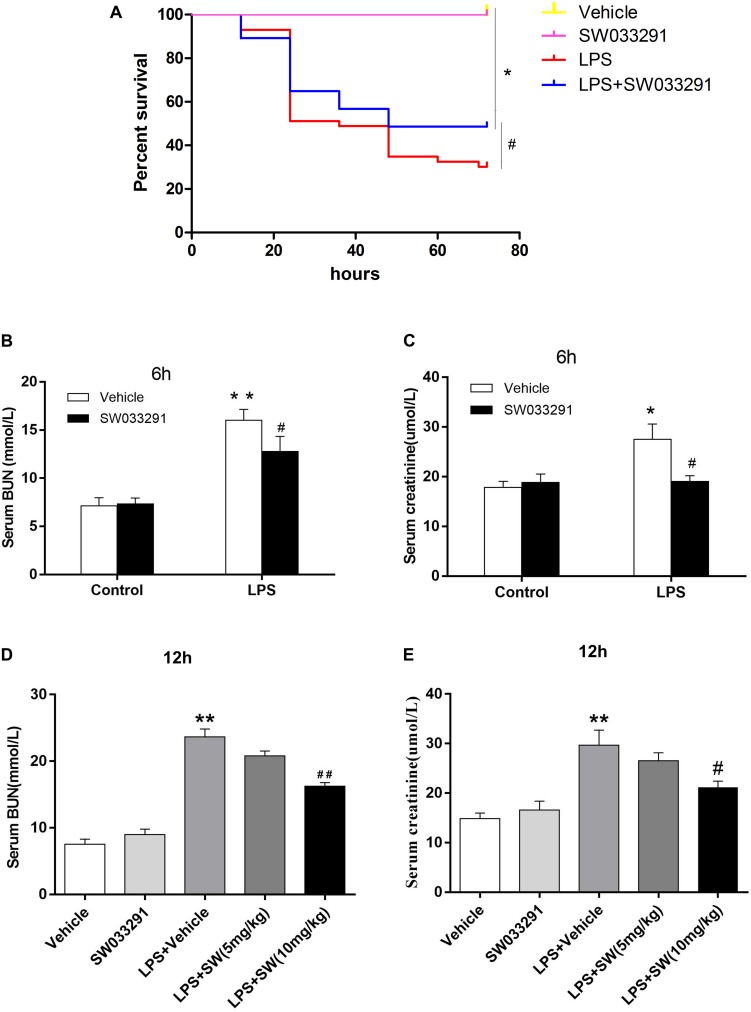
Effect of SW033291 treatment on the survival rate and renal function in mouse sepsis model. **(A)** Effect of SW033291 (10 mg/kg injected twice daily IP for five doses) treatment on the survival of mice in sepsis model. *P < 0.01 (compared with control group); ^#^*P* < 0.05 (compared with LPS group). *n* = 20 in control and SW033291 groups; *n* = 43 in LPS group; *n* = 37 in LPS + SW033291 group; **(B,C)** Serum levels of BUN and Cr in mice with LPS-induced AKI after 6 h of SW033291 (10 mg/kg) treatment (*n* ≥ 6). **(D,E)** Serum levels of BUN and Cr in mice with LPS-induced AKI after 12 h of SW033291 (5 mg/kg or 10 mg/kg) treatment (*n* ≥ 6). *P < 0.05, **P < 0.01 (compared with control group); ^#^*P* < 0.05, ^##^*P* < 0.01 (compared with LPS group). Groups were compared by Two-Way ANOVA. Data are mean ± SD.

**FIGURE 4:**
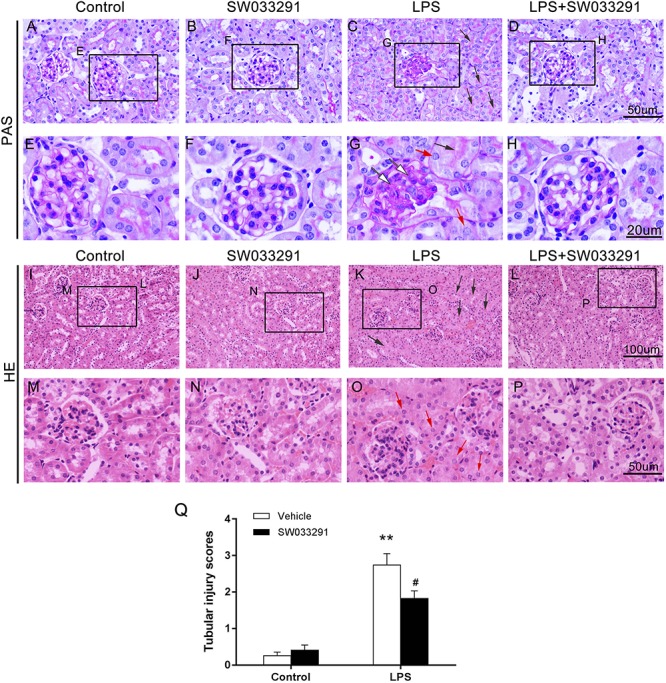
Effect of SW033291 treatment on renal morphologic changes of AKI mice induced by LPS. **(A–D)** PAS staining (12 h) (400×); **(E–H)** Partial enlargement of PAS staining; (*n* = 3) **(I–L)** HE staining (12 h) (200×); **(M–P)** Partial enlargement of HE staining. (*n* = 3) The red arrow ([QSIImage]) indicates renal tubular epithelial cell edema; the black arrow ([QSIImage]) indicates tubular lumen shrinkage; the white arrow ([QSIImage]) indicates hyperplastic glomerular mesangial and thickened basement membrane. **(Q)** Shows the kidney tubular injury scores in mice with LPS-induced AKI after SW033291 (10 mg/kg) treatment, as described in section Materials and Methods (*n* = 3). **P < 0.01 (compared with control group); ^#^*P* < 0.05 (compared with LPS group). Data are mean ± SD.

SW033291 treatment enhanced the survival rate of LPS-treated mice from 30 to 49% (Figure 3A). Meanwhile, renal function assay showed that BUN and Cr levels were significantly increased after 6 h of LPS treatment compared with the control group and peaked at 12 h, whereas the BUN and Cr levels were significantly decreased after SW033291 treatment (Figures 3B–E). The decline in the BUN and Cr levels of mice with 10 mg/kg SW033291 treatment is more obvious than 5 mg/kg dose at 12 h (Figures 3D,E). HE staining showed that there were no significant pathological lesions in the kidneys of the control and SW033291 group mice. In LPS-treated group, the tubular epithelial cells of the mice were edematous with larger cellular volume, vacuolar degeneration and narrowed lumen of the renal tubules. It was also observed that the glomerular structure was disordered, accompanied by infiltration of scattered inflammatory cells and narrowing of the renal capsule. These pathological lesions were evidently alleviated after SW033291 treatment (Figures 4I–Q). PAS staining confirmed above findings in renal morphology (Figures 4A–H). These data indicated a protective effect of SW033291 against LPS-induced AKI.

### SW033291 Treatment Alleviated the Apoptosis of Renal Cells in LPS-Induced AKI Mice

**FIGURE 5:**
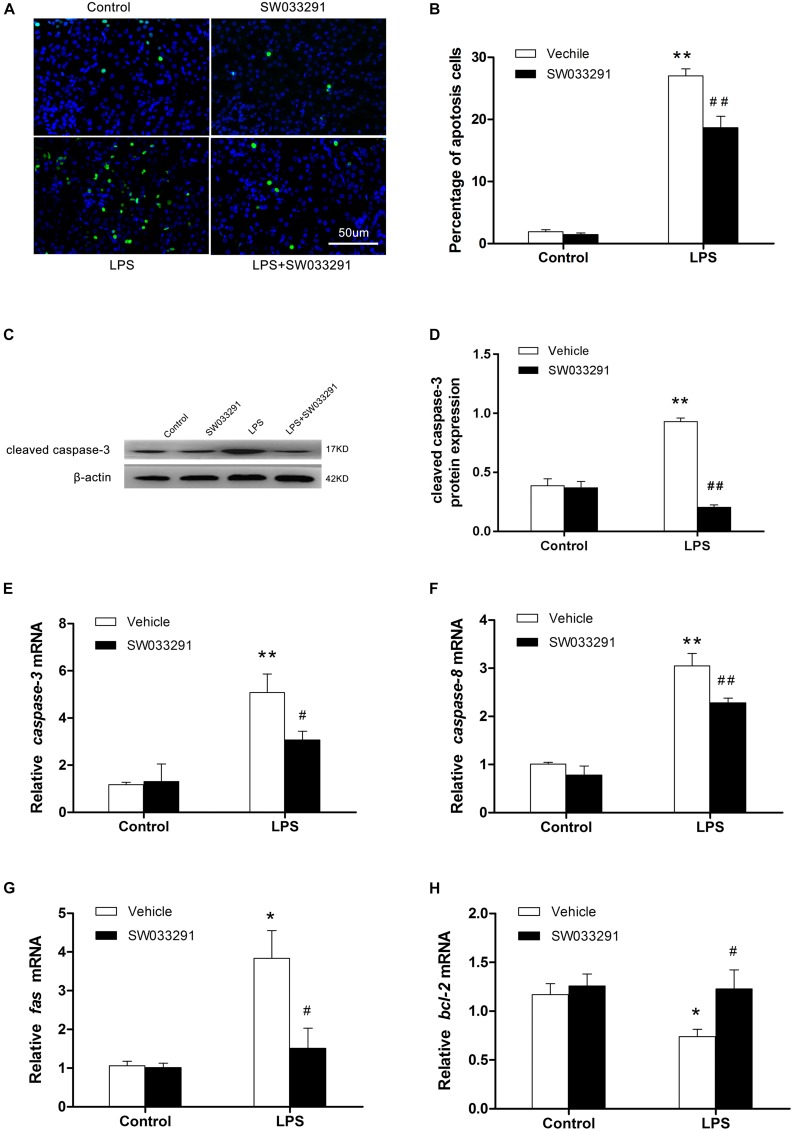
SW033291 treatment alleviated the apoptosis of renal cells in LPS-induced AKI mice. **(A)** TUNEL staining shows the apoptotic renal cells after 12 h of SW033291 (10 mg/kg) treatment (400×). Scale bar: 50 μm. **(B)** Percentage of apoptotic cells (*n* = 6). **(C)** Western blot analysis of cleaved caspase-3 in each group after 12 h of SW033291 (10 mg/kg) treatment. **(D)** Density ratio of cleaved caspase-3/β-actin in each group (*n* = 3). **(E–H)** Real-time PCR was used to detect the apoptotic genes of caspase-3, caspase-8, Fas, and Bcl-2 (*n* = 6). *P < 0.05, **P < 0.01 vs. CON; ^#^*P* < 0.05, ^##^*P* < 0.01 vs. LPS. Data are mean ± SD.

TUNEL assay showed enhanced renal cell apoptosis after intraperitoneal injection of LPS for 12 h in mice, but the apoptosis level was decreased significantly after SW033291 therapy (Figures 5A,B). After LPS stimulation, apoptosis-related protein cleaved-caspase-3 levels increased evidently, whereas SW033291 treatment showed a significant reduction of cleaved-caspase-3 protein expression levels (Figures 5C,D). At the same time, the elevated mRNA levels of Fas, caspase-3, and caspase-8 in kidney after LPS stimulation were all blunted by the pretreatment of SW033291 in line with the restoration of reduced mRNA expression of Bcl-2 (Figures 5E–H). All these data demonstrated an anti-apoptotic role of SW033291 in this experimental setting.

### SW033291 Treatment Increased Autophagic Response in the Kidneys of LPS-Induced AKI Mice

**FIGURE 6:**
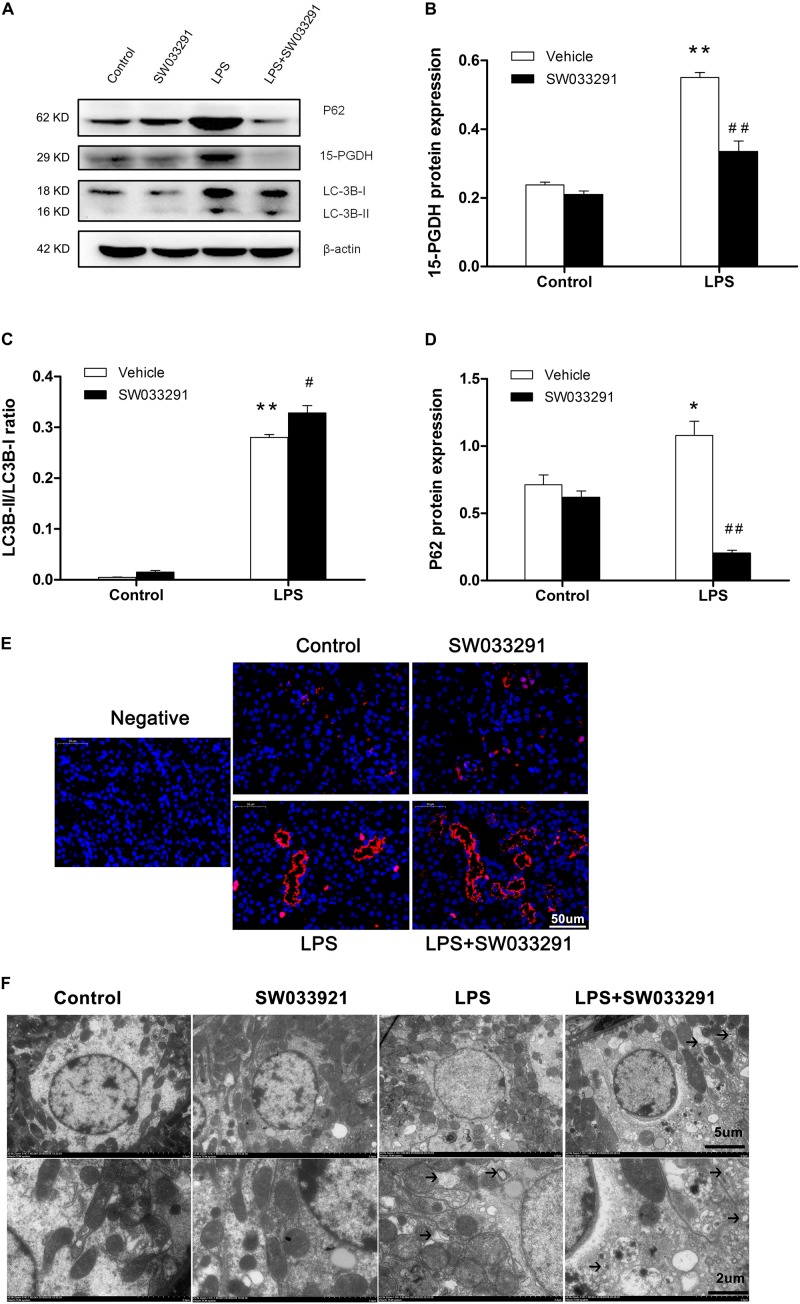
Effect of SW033291 treatment on autophagic response in kidneys of LPS-induced AKI mice. **(A)** Western blotting was used to detect the protein expression of 15-PGDH, LC3B, and P62 in kidney tissues. **(B)** Density ratio of 15-PGDH/β-actin in each group. **(C)** Density ratio of LC3-II/LC3-I in each group. **(D)** Density ratio of P62/β-actin in each group. *P < 0.05, **P < 0.01 vs. CON; ^#^*P* < 0.05, ^##^*P* < 0.01 vs. LPS (*n* = 3). **(E)** Immunofluorescence analysis of kidney tissue from LPS-induced mice with or without SW033291 (10 mg/kg) treatment shows localization of LC3B. Scale bars: 50 μm, 400× (*n* = 3). **(F)** Autophagy level on renal tissue after SW033291 (10 mg/kg) treatment was observed by transmission electron microscope (*n* = 3). Autolysosomes indicated by arrowheads. Scale bars: 5 μm, 2 μm. Data are mean ± SD.

As shown in Figures 6A,B, the expression of 15-PGDH protein was significantly upregulated by LPS treatment. In contrast, SW033291 treatment significantly suppressed 15-PGDH expression in LPS-treated mice. During the development of autophagy, cytosolic soluble LC3 (i.e., LC3-I) is converted to membrane-type LC3 (i.e., LC3-II) and localized on the autophagosome membrane. After LPS stimulation, the LC3-II/LC3-I ratio was significantly increased along with the enhanced P62 protein compared to the control group. Interestingly, SW033291 treatment resulted in a greater increment of the LC3-II/LC3-I ratio after stimulation with LPS (Figures 6A–D). By immunofluorescence, we further confirmed the regulatory role of SW033291 for LC3B in kidneys of AKI mice (Figure 6E). Transmission electron microscopy showed that SW033291 treatment evidently enhanced the number of autophagic vacuoles in kidney cells after LPS stimulation, which further confirmed a promoted autophagic response (Figure 6F).

### SW033291 Treatment Ameliorated Oxidative Damage in the Kidneys of LPS-Induced AKI Mice

**FIGURE 7:**
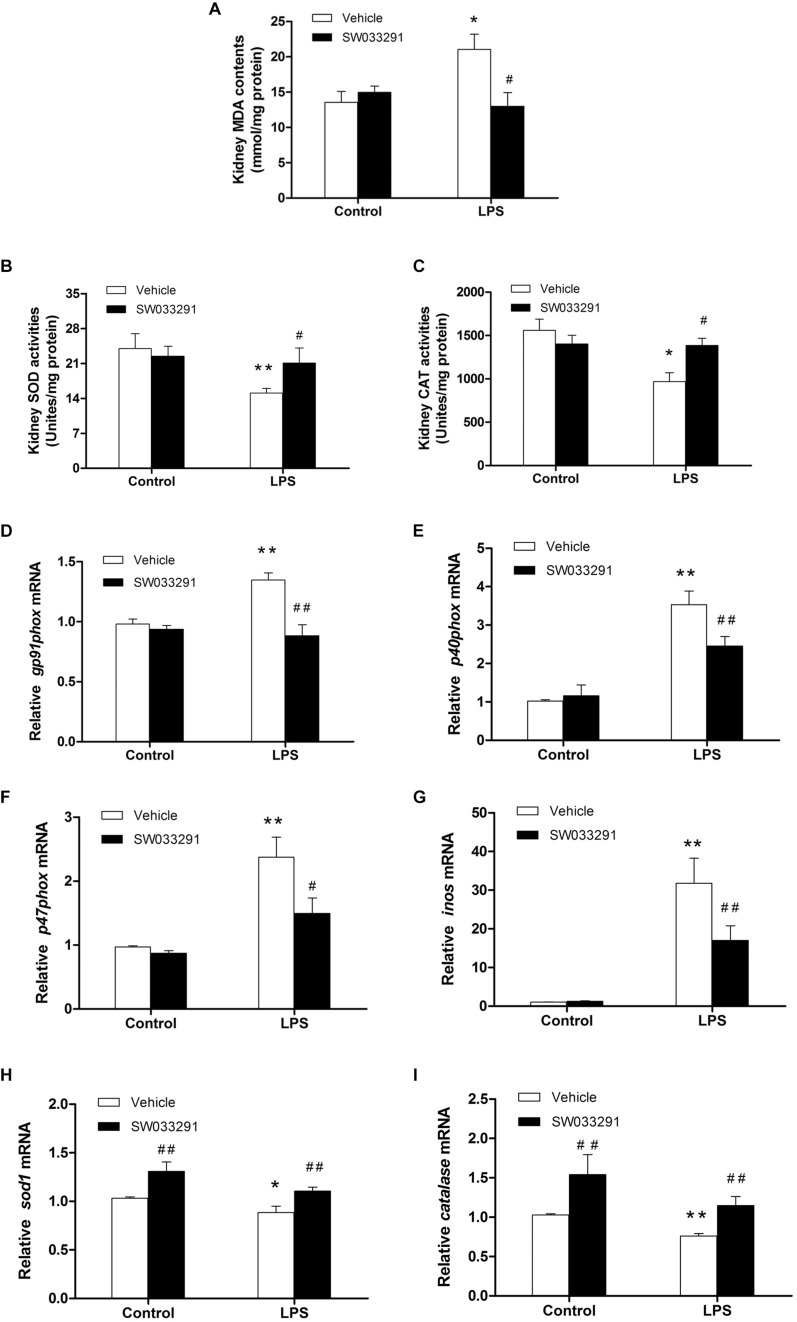
Renal MDA level and the activity of SOD and CAT after SW033291 treatment. **(A)** Renal MDA level. **(B)** Renal SOD activity. **(C)** CAT activity. *P < 0.05, **P < 0.01 vs. CON; ^#^*P* < 0.05, ^##^*P* < 0.01 vs. LPS (*n* ≥ 6). **(D–F)** Effect of SW033291 (10 mg/kg) treatment on the mRNA expression of NADPH oxidase subunits of gp91phox, p40phox, and p47phox in kidneys. **(G)** iNOS mRNA level. **(H,I)** Expression of antioxidant genes of SOD1 and catalase in the kidneys after SW033291 (10 mg/kg) treatment. *P < 0.05, **P < 0.01 vs. CON; ^#^*P* < 0.05, ^##^*P* < 0.01 vs. LPS; (*n* = 6). Data are mean ± SD.

As shown in Figures 7A–C, compared with the control group, the MDA level in the kidney tissue of the LPS group was significantly increased, while the activity of antioxidant enzymes of SOD and CAT was evidently suppressed. Importantly, SW033291 treatment significantly decreased the MDA level and enhanced the activity of SOD and CAT in the kidneys of AKI mice (Figures 7A–C). In agreement with above findings, the mRNA levels of kidney NADPH oxidase subunits gp91phox, p40phox, and p47phox were strikingly upregulated after 12 h LPS challenge compared with the control group, which was significantly blunted after SW033291 treatment (Figures 7D–F). Similarly, the upregulated mRNA expression of iNOS was also reduced by the pretreatment of SW033291 (Figure 7G). Furthermore, we confirmed that the reduction of antioxidant genes of SOD1 and catalase in kidneys after a 12 h injection of LPS was significantly reversed by SW033291 (Figures 7H,I).

### Effect of SW033291 Treatment of Inflammatory Response in the Kidneys of LPS-Induced AKI Mice

**FIGURE 8:**
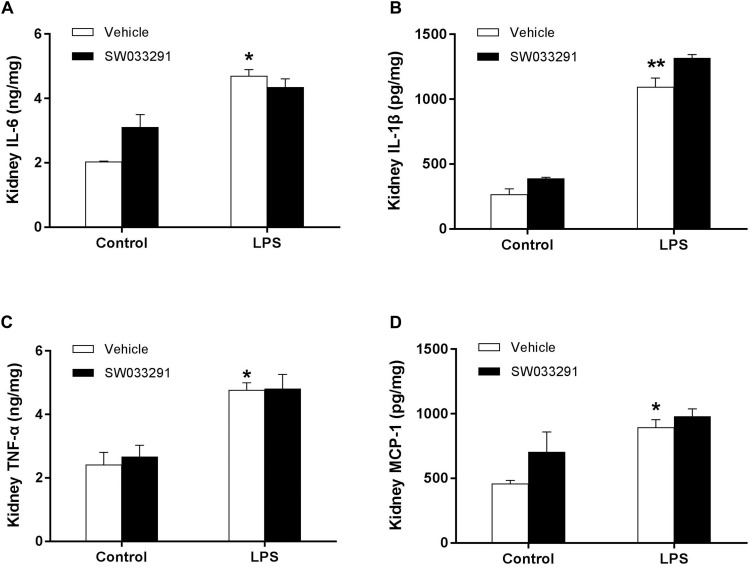
Effect of 15-PGDH inhibition on the release of inflammatory cytokines in kidneys of AKI mice. **(A)** IL-6. **(B)** IL-1β. **(C)** TNF-α. **(D)** MCP-1. *P < 0.05, **P < 0.01 (compared with control group); (*n* = 6). Results were analyzed with Two-Way ANOVA. Data are mean ± SD.

It has been shown that inflammation is a pathogenic factor of AKI. Meanwhile, prostaglandin E2 is a well-established inflammatory mediator. We evaluated whether inflammation was involved in the role of SW033291 in LPS-induced AKI. As shown in Figure 8, LPS treatment significantly enhanced the release of IL-6, IL-1β, TNF-α, and MCP-1 in the kidneys, while SW033291 treatment had no effect on the release of these inflammatory factors. These data suggested that the beneficial role of SW033291 in LPS-induced AKI could be independent of the inflammation.

## Discussion

In the present study, LPS intraperitoneal injection model has been successfully established for septic AKI research according to previous studies. Animals develop a series of systemic inflammation response that mimic clinical sepsis patients (Wu X. et al., 2007; Doi et al., 2009; Gong et al., 2016). The exact mechanisms of sepsis-induced AKI are complex and controversial. Various factors have been known to contribute to the pathologic process of LPS-induced AKI, such as renal cell apoptosis, oxidative stress, and endotoxin-induced complex inflammation (Zarjou and Agarwal, 2011; Prowle and Bellomo, 2015; Shum et al., 2016).

15-PGDH is a key enzyme that expressed widely in mammalian tissues, such as lung, placenta, and kidney, and is mainly localized in the proximal tubules of renal cortex and the outer medullary thick ascending limb (Yao et al., 2008; Liu et al., 2014). In this study, for the first time we discovered that the expression of 15-PGDH protein was increased in kidneys of LPS-stimulated mice and that it was mainly localized in the cytoplasm of renal tubular epithelial cells in renal cortex and outer medulla. More importantly, we also found that the blockade of 15-PGDH could significantly alleviate the kidney damage and contribute to the survival rate of endotoxemic mice. There may be a negative correlation between 15-PGDH expression and kidney function and histology. Therefore, inhibition of 15-PGDH expression may be a potential target for relieving kidney damage. Studies have shown that the renal protective effect is shown by the reduction of apoptosis level in renal tubular epithelial cells in septic AKI (Lerolle et al., 2010; Wei et al., 2010). It has been documented that the apoptosis-associated genes, including Bcl-2, Bax, caspase-3, caspase-8, FasL, and Fas, are closely related to renal injury (Messaris et al., 2008; Lerolle et al., 2010). Our research is consistent with the literature, evidenced by the reduction of renal cell apoptosis in LPS-treated AKI mice after inhibition of 15-PGDH. Therefore, pharmacologic blockade of 15-PGDH may protect against kidney cells apoptosis and kidney injury and then contribute to the survival rate of endotoxemic mice. In agreement with our findings, previous studies have shown that the inhibition of 15-PGDH can promote tissue repair and regeneration after bone marrow transplantation, colitis and partial hepatectomy (Zhang et al., 2015), which also indicate that, in addition to alleviating kidney damage, inhibition of 15-PGDH may affect other organs, thereby improving survival rates in mice.

The LC3-II/LC3-I ratio is usually used to estimate the level of autophagy (Dai et al., 2019; McCormick et al., 2019). Increased levels of LC3-II and decreased levels of p62/SQSTM1 are typically indicative of increased autophagic flux (Klionsky et al., 2016). In the present study, SW033291 treatment promoted LPS-induced autophagy. Studies have shown that autophagy is activated during AKI and protects kidney function (Suzuki et al., 2008; Turkmen et al., 2011). Li et al. (2017) found that increased autophagy could reduce the apoptosis of renal tubular epithelial cell after the occurrence of AKI induced by LPS. Therefore, autophagy may play a protective role in septic AKI via inhibiting apoptosis. These results suggest that inhibition of 15-PGDH may reduce the renal cells apoptosis, possibly related to autophagy.

One of the most important antioxidant enzymes, SOD can catalyze the conversion of harmful superoxide radicals to reduce the oxidative cell damage (Noor et al., 2002; Armogida et al., 2012). CAT can remove hydrogen peroxide and protect cells from damage. Thus, it was possible that inhibition of 15-PGDH reduced the production of oxygen free radicals in kidneys during endotoxemia and relieved the level of lipid peroxidation in the kidney, thereby attenuating the kidney injury. In this study, we also investigated the effect of 15-PGDH on NADPH oxidase subunits in the kidneys of mice treated with LPS. The results indicated that the SW033291 intervention could counteract the upregulation of the NADPH oxidase subunits gp91phox, p40phox, and p47phox induced by LPS. In addition, iNOS may be involved in 15-PGDH-mediated regulation of oxidative damage in AKI caused by LPS. A large amount of literature has indicated that RNS produced by iNOS in the kidney during sepsis could promote I/R-induced renal tubular injury, which was a key reason of AKI during sepsis (Walker et al., 2000; Noiri et al., 2001; Viñas and Sola, 2006; Wu and Mayeux, 2007). It has been shown that iNOS-derived NO/RNS may play a role in the pathogenesis of LPS-induced renal injury (Wang et al., 2003; Cuzzocrea et al., 2006). Selective inhibition of iNOS can reduce RNS production and improve sepsis-induced renal impairment (Heemskerk et al., 2009). The present study also found that inhibition of 15-PGDH could effectively reverse the downregulation of the antioxidation enzymes SOD1 and catalase induced by LPS. Antioxidants can reduce AKI caused by sepsis (Campos et al., 2012; Li et al., 2014). Therefore, 15-PGDH may promote the oxidative damage during LPS-induced AKI, and its mechanism may be related to the upregulation of NADPH oxidase subunits and iNOS and downregulation of the expression of antioxidant enzymes in the kidney. In oxaliplatin-resistant HT29 cells, PGE_2_ levels were significantly elevated, with elevated COX-2 and reduced 15-PGDH expression. The COX-2/PGE2/EP4 signaling axis play an important role on oxaliplatin resistance via regulation of oxidative stress (Huang et al., 2019). In previous studies, injection of SW033291 [10 mg per kg of weight (mg/kg)] induced a significant increase of PGE_2_ and Prostaglandin D2 (PGD2) levels, while PGF2a levels were mostly unaltered (Zhang et al., 2015). 15-PGDH catalyze the conversion of 15-hydroxy group of PGE_2_ into 15-keto-PGE2 (15k-PGE2), which further catalyzed by prostaglandin reductase 2 (PTGR2). Exogenous 15k-PGE2 treatment or PTGR2 knockdown yielded the antioxidative transcription factor increase in LPS-stimulated RAW264.7 cells (Chen et al., 2018). Therefore, we speculate that 15-PGDH may regulate oxidative stress via COX-2/PGE2/EP4 signaling axis. However, other prostaglandins, such as PGD2, may play an important role, and the precise mechanism needs to be further investigated.

We also demonstrated that SW033291 treatment had no effect on the release of IL-6, IL-1β, TNF-α, and MCP-1. The effect of LPS is mediated by Toll-like receptor 4 (TLR-4) and the release of inflammatory cytokines, such as TNF-a (initiator of inflammatory response). Cunningham et al. (2004) found that LPS can induce systemic TNF-α releasing, promoting inflammatory cell infiltration and tubular cell apoptosis in septic AKI. These results indicate that 15-PGDH has little effect on the inflammatory response during LPS-induced AKI.

## Conclusion

**FIGURE 9:**
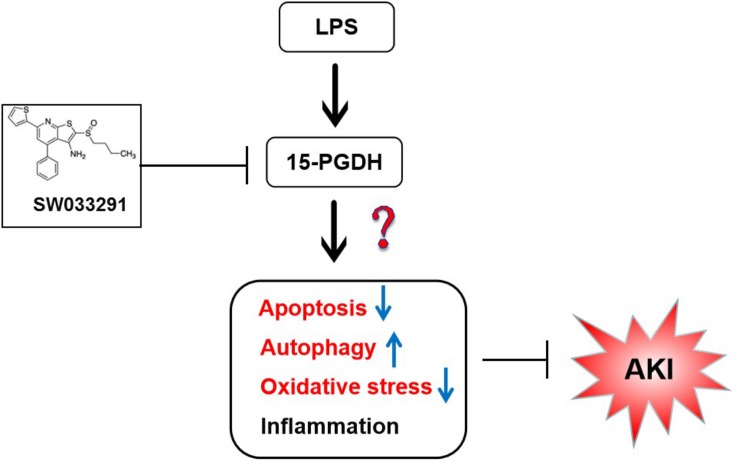
The summary flow diagram.

In summary, 15-PGDH expression was upregulated in kidneys of LPS-stimulated AKI mice, contributing to LPS-induced AKI. And such an effect may be related to apoptosis, autophagy, and oxidative damage (Figure 9). However, it is unclear whether there is interaction between apoptosis, autophagy, and oxidative damage, which needs to be further investigated. Our study provided new insights for the prevention and treatment of sepsis-related AKI.

## Data Availability Statement

The raw data supporting the conclusions of this article will be made available by the authors, without undue reservation, to any qualified researcher. Requests to access the datasets should be directed to YL, liu1977ying@126.com.

## Ethics Statement

The animal study was reviewed and approved by the Experimental Animal Ethics Committee of Central South University.

## Author Contributions

SM, YL, HZ, and XX conceived and designed the experiments. SM, CL, JZ, TL, and YX executed the experiments and analyzed the samples. SM, CL, YL, XW, and CW analyzed the data. SM wrote the first version of the manuscript. All authors interpreted the data, critically revised the manuscript, and approved the final version of the manuscript.

## Conflict of Interest

The authors declare that the research was conducted in the absence of any commercial or financial relationships that could be construed as a potential conflict of interest.

## Abbreviations

AKI: acute kidney injury
LPS: lipopolysaccharide
15-PGDH: 15-hydroxyprostaglandin dehydrogenase
MDA: malondialdehyde
SOD: superoxide dismutase
CAT: catalase
real-time PCR: real-time reverse transcriptase polymerase chain reaction
IL-6: interleukin-6
IL-1 β: interleukin 1 beta
TNF- α: tumor necrosis factor- α
MCP-1: monocyte chemoattractant protein-1
ROS: reactive oxygen species
RNS: reactive nitrogen
PGE_2_: Prostaglandin E_2_
PGD2: Prostaglandin D2
PTGR2: prostaglandin reductase 2
RA: rheumatoid arthritis
TLR-4: Toll-like receptor 4.
